# Iatrogenic Brachial Plexus Injuries Evaluated in an Electrodiagnostic Lab

**DOI:** 10.3390/neurolint18090166

**Published:** 2026-08-27

**Authors:** Lisa B. E. Shields, Vasudeva G. Iyer, Smita Ghare, Christopher B. Shields

**Affiliations:** 1 Norton Neuroscience Institute, Norton Healthcare, Louisville, KY 40202, USA; smita.ghare@nortonhealthcare.org (S.G.); cbshields1@gmail.com (C.B.S.); 2 Neurodiagnostic Center of Louisville, Louisville, KY 40245, USA; pavaiyer@gmail.com

**Keywords:** neurology, brachial plexus injury, shoulder surgery, coronary artery bypass, iatrogenic, electrodiagnostic study

## Abstract

**Background/Objectives:** Iatrogenic brachial plexus injuries (BPI) are known to occur following a host of surgeries, most often during procedures at the shoulder and cardiac procedures with a median sternotomy. The primary mechanisms include overstretching of the brachial plexus, direct trauma, or compression by hematoma or seroma. Our objective was to determine the pattern of iatrogenic BPI in patients referred to us for electrodiagnostic (EDX) evaluation. **Methods:** This is a review of 52 patients who were referred to our Neurodiagnostic Center for an iatrogenic BPI over a 16-year (9 July 2010–17 June 2026) period. All patients underwent a clinical examination and EDX studies. **Results:** The most frequent etiologies of the iatrogenic BPI were shoulder surgery in 28 [53.8%] patients and coronary artery bypass graft procedures with a median sternotomy in 13 [25.0%] patients. The topography of brachial plexus involvement was trunks in 22 (42.3%) and cords in 12 (23.1%). Ten (19.2%) patients had a pan-plexopathy, and the remaining eight (15.4%) patients had multiple sites involving the trunks, cords, and branches. The majority (48 [92.3%]) of patients sustained a stretch injury of the brachial plexus, and four (7.7%) sustained a direct injury. A total of 48 (92.3%) patients had reduced motor unit potential recruitment and/or increased polyphasic motor units on needle EMG, and a similar number had an absence of or low amplitude sensory nerve action potentials. **Conclusions:** Surgeons should take appropriate precautions to avoid BPI during many surgical procedures especially those of the shoulder and cardiac procedures with a median sternotomy. EDX studies are valuable in detecting and localizing an iatrogenic BPI as well as assessing reinnervation. Preoperative positioning limiting excessive shoulder abduction as well as intraoperative neuromonitoring are measures that may reduce the likelihood of an iatrogenic BPI.

## 1. Introduction

Brachial plexus injuries (BPI) comprise 1.2% of polytrauma and may occur in a variety of settings, including obstetrics, trauma (motor vehicles accidents), iatrogenic, or radiation-induced [1]. Iatrogenic BPI represent debilitating injuries that affect the upper extremity and may ensue following several operative procedures, including shoulder surgery [2,3], cardiac surgery with a median sternotomy [2,4,5], prolonged spinal surgery [6,7], hip arthroplasty [8], vascular access for Extracorporeal Membrane Oxygenation (ECMO) and subclavian vein cannulation [9,10], radical mastectomy [11,12,13], rib resection for thoracic outlet syndrome [2,14], resection or biopsy of a peripheral nerve sheath tumor or excision of lymph nodes at the cervicomediastinal area [2], and improper use of high-thoracic restraints [15]. BPI may also occur following brachial plexus anesthetic (interscalene, supraclavicular, or axillary) block or vessel puncture (during central venous catheterization or axillary angiography) due to direct needle trauma, chemical neurotoxicity, or hematoma/seroma formation [16]. Patients with BPI may experience sensory abnormalities and/or decreased strength of the ipsilateral upper extremity. Approximately 7–10% of brachial plexopathies are iatrogenic [17]. The rate of BPI following shoulder arthroplasty has been reported as 2.3% [3], while the incidence of BPI during open heart surgery with a median sternotomy has been reported to occur between 2 and 38% with lower trunk lesions as the most common [4,18].

Detailed knowledge of the brachial plexus anatomy is imperative to decrease the likelihood of sustaining an iatrogenic injury. Ventral/anterior primary rami of the C5 through T1 spinal nerves comprise the roots of the brachial plexus [19]. The roots merge to form three trunks: superior (C5-6), middle (C7), and inferior (C8-T1). The trunks then divide into six (anterior and posterior) divisions which then form three cords (lateral, posterior, and medial). Five major nerves arise from the cords as terminal branches of the brachial plexus: musculocutaneous from the lateral cord, median from the lateral and medial cords, and ulnar from the medial cord as well as the axillary and radial nerves from posterior cord. The subclavian artery and its derivatives provide blood supply to the brachial plexus.

The primary mechanisms that may cause an iatrogenic BPI include overstretching of the brachial plexus, direct trauma, or compression by hematoma or seroma [7,11]. Shoulder abduction greater than 90 degrees, external rotation of the arm, and posterior shoulder displacement may all be responsible for stretching the brachial plexus [7]. Nerve injury may occur if the nerve is stretched beyond 5–15% of its resting length which results in edema of the nerve, an increase in the intraneural pressure, and a reduction in the perfusion pressure of the nerve fibers [7]. Intraneural blood flow is minimized, leading to ischemia and endoneurial edema.

While case reports and series describing iatrogenic BPI following various surgeries have been reported, a comprehensive analysis of iatrogenic BPI with its numerous etiologies is lacking. In this study, we describe the clinical and EDX findings in patients who were referred to our EMG lab for evaluation of iatrogenic BPI. We also discuss the various causes, preventive measures, and potential treatments of iatrogenic BPI.

## 2. Materials and Methods

Under an Institutional Review Board (IRB)-approved protocol, we performed a 16-year (9 July 2010–17 June 2026) retrospective analysis of patients with an iatrogenic BPI who were referred to our Neurodiagnostic Center for EDX studies. Our American Association of Neuromuscular and Electrodiagnostic Medicine (AANEM)-accredited Neurodiagnostic Center assesses approximately 1000 patients annually referred for EDX studies predominantly by hand, orthopedic, and neurosurgeons. All patients with an iatrogenic BPI underwent a clinical examination and EDX studies. The EDX protocol includes nerve conduction and needle electromyography (EMG) studies. The same electromyographer, a neurologist board-certified in electrodiagnostic medicine and clinical neurophysiology, performed the evaluations of all patients. The mean duration of time between the patients’ surgery when the iatrogenic brachial plexus injury occurred and the EDX study was 8.45 months (Range: 0.92–60.0 months).

### 2.1. Inclusion and Exclusion Criteria

Inclusion criteria were adult patients referred for EDX studies with a diagnosis of iatrogenic BPI. Only patients that exhibited symptoms and signs of brachial plexopathy immediately after the surgical procedure were included. Patients with a delayed onset of brachial plexopathy after surgery were excluded due to the possibility of post-surgical Parsonage-Turner syndrome [20]. Patients with BPI unrelated to a surgical procedure were also excluded.

### 2.2. Metrics Collected

The clinical metrics collected included: (1) demographics, (2) laterality (left, right, or bilateral), (3) type of surgery that caused the iatrogenic BPI; and (4) EDX findings. The localization of the BPI was divided into 3 primary categories: (1) trunk (upper and lower); (2) cord (medial, lateral, and posterior); and (3) pan-plexopathy (all trunks/cords of varying severity based on the distribution of EMG abnormalities). There were also combinations of these categories as well as with the emerging major nerves. The mechanism of BPI was based on the most likely pathogenesis. When a hematoma/seroma was documented by MRI, we considered “compressive etiology”. When the injury followed injections or other procedures in and around the brachial plexus, we considered “direct injury.” When the injury occurs following procedures such as shoulder repair or replacement, it was presumed that there may have been a stretch injury to the brachial plexus.

The EDX studies including motor and sensory conduction as well as needle EMG were performed utilizing the standard protocol in our lab [21]. Study of sensory conduction in the medial and lateral antebrachial cutaneous nerves as well as the topography of denervation of muscles were used for accurate localization of injury to the specific part of the brachial plexus.

### 2.3. Institutional Review Board Approval of Research

The study was conducted according to the guidelines of the Declaration of Helsinki and was approved by the WCG Institutional Review Board. The IRB determined that our study was exempt under 45 CFR 46.104(d4). The IRB number is 20253566, and the Ethic Approval Code is 09152025. The IRB approval date was 15 September 2025.

## 3. Results

### 3.1. Demographics

Of the 52 patients with an iatrogenic BPI, 34 (65.4%) were male, and the mean age was 62 years (Range: 24–81 years) (Table 1). The BPI was on the right side in 27 (51.9%) patients, left side in 24 (46.2%), and bilateral in one (1.9%). A total of 47 (90.4%) patients were right hand dominant. The side of the BPI corresponded to the dominant hand in 31 (59.6%) patients.

### 3.2. Etiology, Localization, and Mechanism of Iatrogenic Brachial Plexus Injuries

**Figure 1:**
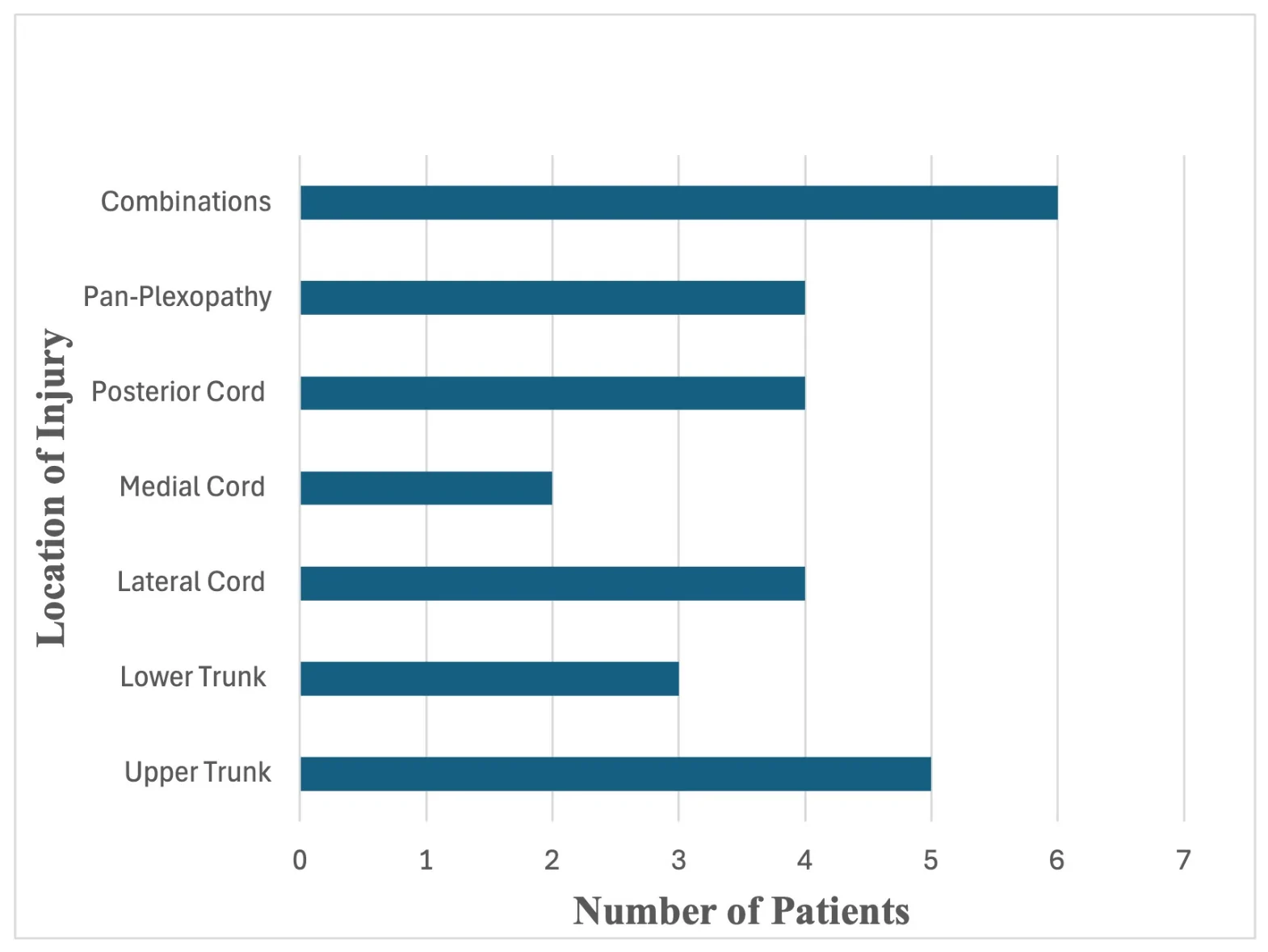
Location of injuries in patients who sustained an iatrogenic brachial plexus injury following shoulder surgery.

**Figure 2:**
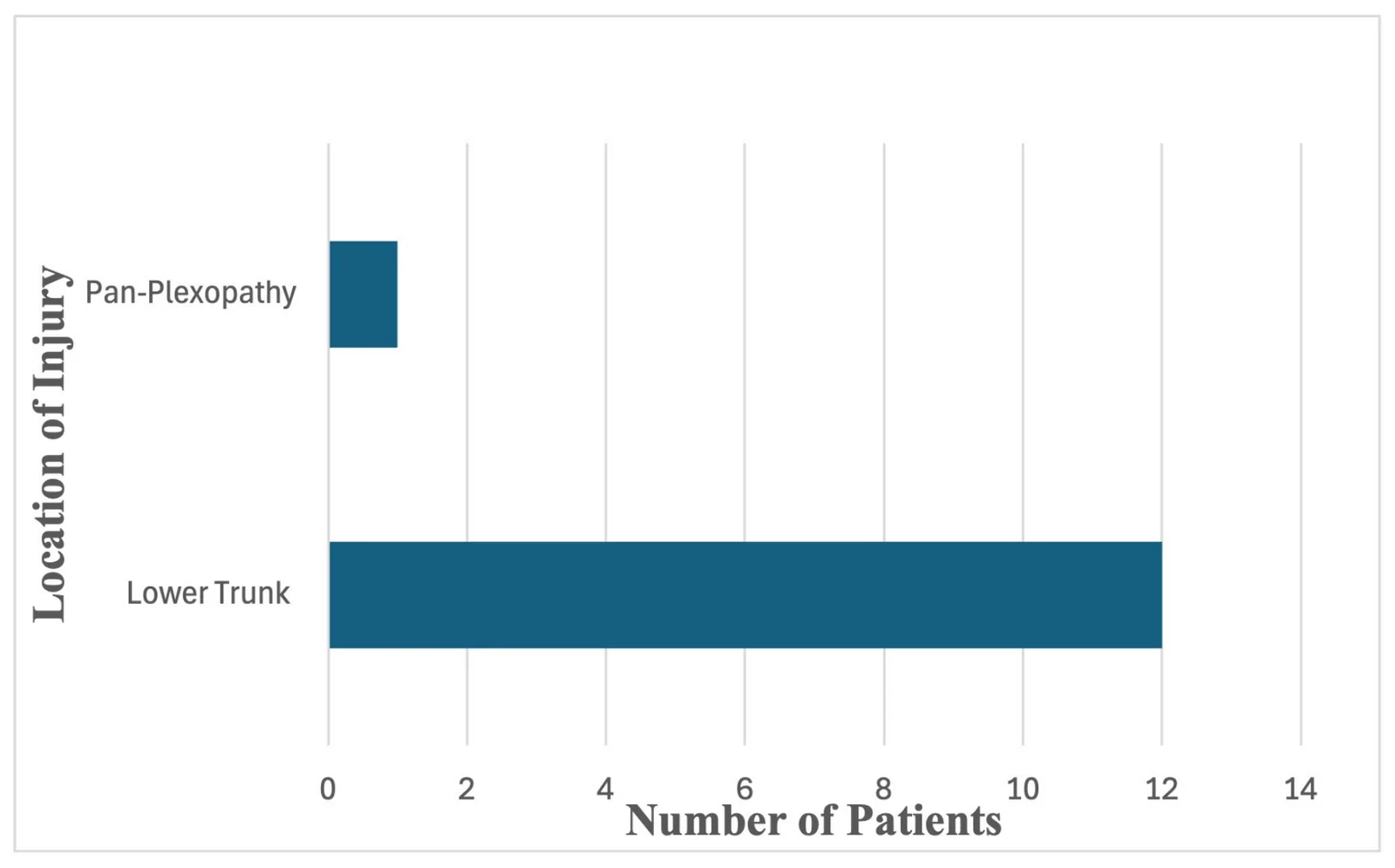
Location of injuries in patients who sustained an iatrogenic brachial plexus injury following coronary artery bypass graft with a median sternotomy.

Iatrogenic BPI was most common with surgical procedures at the shoulder (28 [53.8%] patients) (Figure 1) and coronary artery bypass graft procedures (CABG) with a sternotomy (13 [25.0%] patients) (Figure 2) (Table 2). Eleven (21.2%) patients underwent other types of surgeries that caused the BPI: aortic valve surgery/repair of aortic aneurysm with a sternotomy (two); total hip replacement (one); mastectomy/axillary lymphadenectomy (one); breast reconstruction following mastectomy/axillary lymphadenectomy (one); ECMO (one); port at the infraclavicular region (one); prolonged lumbar spine surgery (one); restraints for a combative patient (one); subclavian artery access in the infraclavicular region (one); and thoracic outlet syndrome surgery (one).

Of the 52 patients with an iatrogenic BPI, 22 (42.3%) had involvement of only the trunk (upper: 5 [22.7%]; lower: 17 [77.3%]) and 12 (23.1%) had involvement of only the cord (medial: 4 [33.3%); lateral: 4 [33.3%]; posterior: 4 [33.3%]) (Table 3). Ten (19.2%) patients had a pan-plexopathy. The remaining eight (15.4%) patients had a combination of two locations, consisting of either trunks, cords, a pan-plexopathy, and/or the emerging median nerve (Table 3). When combining the number of patients who had isolated involvement of the trunk or the cord with those who had two or more localizing areas, 23 had trunk involvement, 17 had cord involvement, and 11 had a pan-plexopathy. Of the 13 patients who underwent cardiac surgery with a median sternotomy, the localization of the BPI was the lower trunk in 12 (92.3%) patients.

The majority (48 [92.3%]) of patients sustained a stretch injury of the brachial plexus, while four (7.7%) experienced a direct injury (Table 4). These latter four patients underwent one of the following procedures: thoracic outlet syndrome surgery, port insertion at the infraclavicular region, subclavian artery access at the infraclavicular region, and ECMO.

### 3.3. Electrodiagnostic Studies

In the 52 patients who underwent EDX study, 48 (92.3%) had features of motor axonal injury in the form of reduced motor unit recruitment and increase in polyphasic motor units and a similar number had sensory axonal injury with an absence of or low amplitude sensory nerve action potentials (Table 5).

## 4. Representative Cases

### 4.1. Case #1

Shoulder surgery: A 60-year-old female presented with severe weakness and numbness of the right upper extremity after undergoing reverse shoulder arthroplasty. Marked weakness of the right deltoid, biceps, pronator teres, brachioradialis, and triceps muscles was observed. Pinprick sensation was lost over the lateral aspect of the upper arm, and the biceps and brachioradialis relaxes were absent. No compound muscle action potential (CMAP) was detected over the deltoid, and a small CMAP was noted over the biceps on stimulation at the Erb’s point. Small amplitude sensory nerve action potential (SNAP) was detected over the superficial radial nerve. There was total denervation of the deltoid, infraspinatus, biceps, extensor carpal radialis longus (ECRL), and brachioradialis muscles. An upper and middle trunk axonal injury was the localization of the iatrogenic BPI. 

### 4.2. Case #2 

Coronary artery bypass surgery: A 78-year-old man complained of weakness, pain, and paresthesia of the left upper extremity after a coronary artery bypass surgery with sternotomy. Weakness of all intrinsic hand muscles as well as FDP, extensor digitorum communis (EDC), and FPL muscles was noted. Pinprick sensation was decreased over the small and ring fingers, ulnar palm, and medial forearm. Low amplitude CMAP over the abductor pollicis brevis (APB) muscle were detected on median nerve and over the abductor digiti minimi (ADM) muscle on ulnar nerve stimulation with diffuse slowing of motor conduction. Sensory potentials of very small amplitudes were detected over the ulnar three digits. The medial antebrachial cutaneous SNAP was absent. There was denervation of the ADM, first dorsal interosseous (FDI), APB, and EDC muscles. The localization was lower trunk plexopathy with axonal injury.

### 4.3. Case #3

Thoracic outlet syndrome surgery: A 30-year-old female reported her hands becoming “cold and stiff” and paresthesia primarily of the small finger. The patient underwent a 1st rib resection for thoracic outlet syndrome on the right. She complained of marked weakness of the hand immediately after she woke up from anesthesia. There was decreased motor unit recruitment and an increase in polyphasic motor units in the APB muscle. The conclusion was severe right lower trunk plexopathy most likely from a direct injury to the plexus.

### 4.4. Case #4 

**Figure 3:**
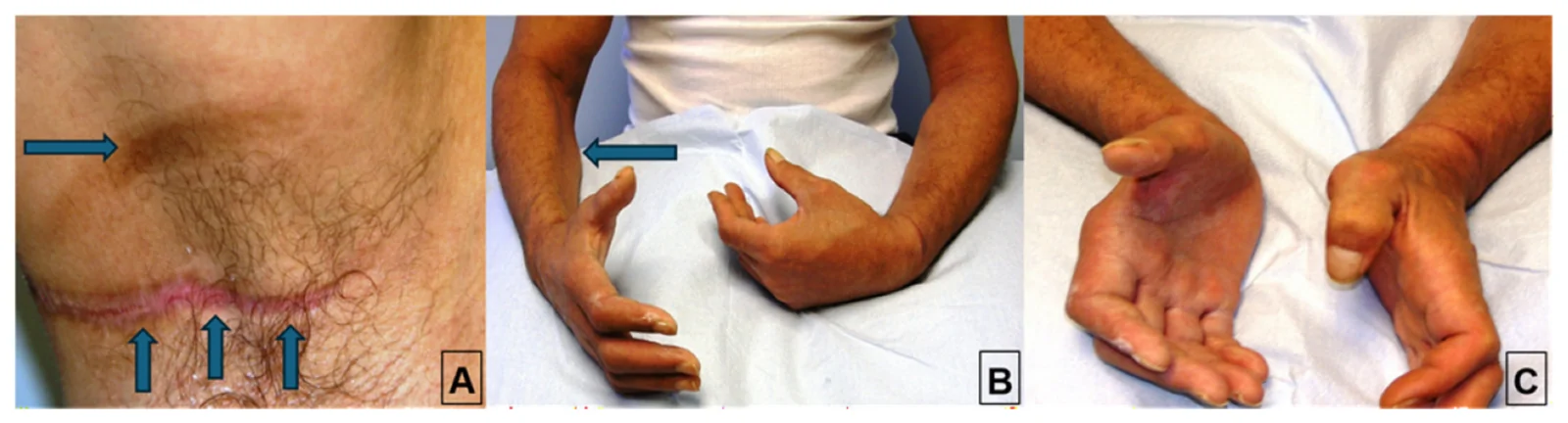
(**A**) Scars from the vest restraint (arrows). (**B**,**C**) Weakness of the flexors of the wrists and all of the digits on the right. Arrow in (**B**) points to atrophy of the pronator and flexors with a normal brachioradialis in the right upper extremity.

Restraints in a combative patient: A 53-year-old male was referred for evaluation of weakness and numbness of the upper extremities bilaterally. He was restless and combative after sustaining a skull fracture and was placed in a vest with wrist restraints (Figure 3A). When he became lucid, he noted significant weakness of the upper extremities and displayed a left 6th cranial nerve palsy with complaints of diplopia. Clinical exam detected marked weakness of all muscles supplied by median and ulnar nerves and loss of pinprick sensation in the distribution of the median, ulnar, and medial antebrachial cutaneous nerve (MABCN) on the right (Figure 3B,C). There was also weakness of muscles innervated by the left ulnar and median nerves with loss of pinprick sensation in the distribution of the ulnar and medial antebrachial cutaneous nerves. EDX studies detected features of complete axonotmesis in the median and ulnar nerves on the right with no SNAP over the median and ulnar nerves and MABCN. There was evidence for partial axonotmesis in the left median and ulnar nerve with no SNAP over the ulnar nerve and MABCN. There was also an additional injury of the left ulnar nerve at the elbow, most likely due to external pressure. The localization included the (1) right medial cord with additional injury to median and ulnar nerves at their origin from the medial cord and (2) the left medial cord with additional injury to the ulnar nerve at elbow. The most likely mechanism was a stretch injury of the right brachial plexus involving the medial cord and the emerging right median nerve. Scarring around the medial cord and the emerging median, ulnar, and medial antebrachial cutaneous nerves in the axilla was noted intraoperatively, and an external neurolysis was done. At 9 months significant reinnervation was noted.

### 4.5. Case #5 

**Figure 4:**
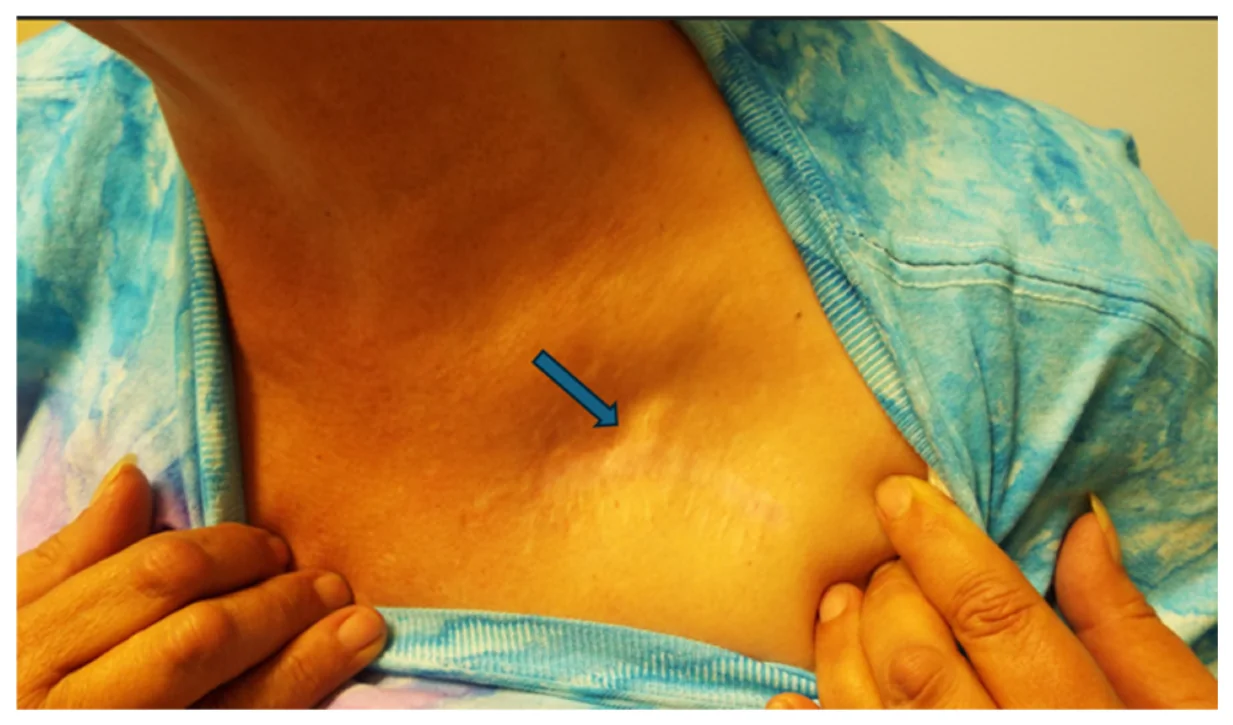
Subclavian artery access in the infraclavicular region (arrow) in a patient who underwent an axillo-femoral bypass surgery.

Subclavian artery access: A 54-year-old female who underwent a subclavian artery access procedure in the infraclavicular region for a left axillo-femoral bypass in a 7-h surgery (Figure 4). A tunneler was placed from the subclavian incision to the femoral incision, and the subclavian artery was clamped as it became the axillary artery. The patient had a pan-plexopathy on the left which most likely resulted from direct injury of the brachial plexus.

## 5. Discussion

While several cases have been reported describing iatrogenic BPI after surgery at numerous anatomical locations, to our knowledge only one study in the literature reported a large series of patients with iatrogenic BPI [2]. In Dengler and colleagues’ study of 14 patients who sustained an iatrogenic BPI, the most common etiology was shoulder surgery (rotator cuff fixation, arthroplasty, and repositioning of a clavicle fracture) in seven (50%) patients, followed by resection or biopsy of a peripheral nerve sheath tumor (three), lymph node at the cervicomediastinal areas (two), transaxillary rib resection (one), and sternotomy (one) [2]. All patients presented with significant impairment of motor function of the upper extremities, and 11 patients had neuropathic pain. Treatment of the BPI included nerve grafting (nine), neurolysis (four), and nerve transfer (one), with improved symptoms afterwards in 11 patients. These authors recommend early referral to specialized peripheral nerve centers to optimize patient outcomes [2].

Our study concurs with that of Dengler and colleagues with respect to shoulder surgery as the most common procedure that caused an iatrogenic BPI. In the current study, more than half of the patients with BPI underwent shoulder surgery and 25% underwent cardiac surgery with a median sternotomy. In the latter group, the localization of the BPI was the lower trunk in 92% of patients. Several mechanisms have been postulated for a BPI during cardiac surgery with a median sternotomy. When the sternum is retracted, the retroclavicular space narrows which can potentially cause stretching and compression of the lower trunk between the clavicle and the rotated 1st rib [22]. This is further worsened by the abduction and external rotation of the arm with posterior displacement of the shoulder. Another mechanism is fracture of the 1st rib at the costovertebral junction (due to retraction of the rib cage) which may injure the C8 anterior primary ramus [5]. More than 90% of the patients with an iatrogenic BPI sustained a stretch injury of the brachial plexus, while less than 10% had a direct injury. A total of 92.3% of patients had sensory and motor abnormalities detected on EDX studies.

Several measures may be implemented to decrease the likelihood of occurrence of an iatrogenic BPI (Table 6). In 2018 the American Society of Anesthesiologists Task Force on Prevention of Perioperative Peripheral Neuropathies updated their report from 2000, emphasizing appropriate positioning strategies to reduce perioperative brachial plexus neuropathy [23]. Arms should not be kept in more than 90 degrees abduction while placed in the supine position. Intraoperative neuromonitoring is a valuable tool to minimize the risk of BPI [24,25]. This technique provides real-time feedback during surgeries and detects nerve distress from excessive stretching, compression, or surgical manipulation. This permits the surgeon to change the patient position or surgical approach to decrease the likelihood of permanent damage.

Clinical examination and EDX studies are valuable in detecting an iatrogenic BPI [11,26]. EDX studies determine the location of the injury, differentiate conduction block from axonal injury, assess the severity of injury, and provide prognostic clues [26]. Treatment of an iatrogenic BPI depends on the location and type (axonal versus conduction block) of the injury, degree of damage, and interval between the injury and repair [26]. Prevention involves protecting the upper extremity from ongoing traction, preserving shoulder range of motion, and establishing alternative approaches [26]. Most patients with BPI are referred for EMG studies if no clinical recovery is notable after 2-3 months. If early signs of reinnervation are noted, surgical intervention is deferred. If no reinnervation is seen in the denervated proximal muscles on serial EMG studies, surgical intervention is considered. Depending upon the extent and severity of the injury, the surgical repair may involve nerve grafts as well as, where feasible, nerve transfers to facilitate early reinnervation of distal muscles such as the intrinsic hand muscles. Nerve transfers involve “coapting a healthy expandable donor nerve or fascicle to a denervated recipient in order to restore function to the recipient end organ” [27]. A typical example is connecting fascicles of the intact ulnar nerve to the musculocutaneous nerve to achieve reinnervation of the biceps muscle in cases of axonotmesis of the lateral cord or the upper trunk. Nerve transfers using this technique have shown good functional outcomes in BPI [27]. While patients often attain improvement following an iatrogenic BPI, others may experience permanent deficits [11].

### Strengths and Limitations

The strength of the present study is that it describes the largest number of patients to date with an iatrogenic BPI and provides data on the anatomical pattern of injury and type of surgical procedures most likely to cause BPI. It also highlights the role of EDX studies in localizing the site of injury within the brachial plexus and determining the severity of injury. Since the EDX studies were conducted by the same electromyographer, there was no possibility for inter-observer error. Our lab is accredited by AANEM with exemplary status, and the protocols were approved by AANEM. Limitations of this study include its retrospective nature and lack of follow-up as most patients were only evaluated on one occasion at our Neurodiagnostic Center. Another limitation is the potential selection bias since most patients were referred by hand, orthopedic, and neurosurgeons. The 16-year recruitment period in our study may also introduce heterogeneity due to changes in surgical and diagnostic practices over time.

## 6. Conclusions

In this study of a large cohort of patients with iatrogenic BPI, we discuss the type of surgical procedures that often caused injury, the pattern of injury, and possible mechanism. Surgeons should be aware that BPI may occur during a host of surgeries, most often shoulder surgery and cardiac procedures with a median sternotomy. Perioperative positioning and intraoperative precautions to avoid stretch injury to the brachial plexus as well as intraoperative neuromonitoring are measures that can potentially minimize BPI.

## Figures and Tables

**Table 1:** Demographics of Iatrogenic Brachial Plexus Injuries.

Metrics	Number of Patients (*n* = 52)
Age	Mean: 62.0 years (Range: 24–81 years)
Gender	
Male	34 (65.4%)
Female	18 (34.6%)
Side of symptoms	
Right	27 (51.9%)
Left	24 (46.2%)
Bilateral	1 (1.9%)
Symptoms correspond to dominant hand	
Yes	31 (59.6%)
No	21 (40.4%)

**Table 2:** Type of Surgery and Brachial Plexus Injury Localization.

Metrics		Number of Cases (*n* = 52)
Surgery Type	Brachial Plexus Localization	
Shoulder		28 (53.8%)
	Upper trunk	5 (17.8%)
	Lateral cord	4 (14.3%)
	Posterior cord	4 (14.3%)
	Pan-plexopathy	4 (14.3%)
	Lower trunk	3 (10.7%)
	Medial cord	2 (7.1%)
	Upper and middle trunks	1 (3.6%)
	Medial and posterior cords	1 (3.6%)
	Lateral and posterior cords	1 (3.6%)
	Medial cord and median nerve	2 (7.1%)
	Lateral cord and median nerve	1 (3.6%)
Coronary artery bypass graft with sternal split		13 (25.0%)
	Lower trunk	12 (92.3%)
	Pan-plexopathy	1 (7.7%)
Others *		11 (21.2%)
	Pan-plexopathy	5 (45.4%)
	Lower trunk	2 (18.2%)
	Medial cord	2 (18.2%)
	Pan-plexopathy (B)	1 (9.1%)
	Medial cord and median nerve (R)	1 (9.1%)

B: bilateral; R: right. * Others: aortic valve surgery/repair of aortic aneurysm with sternal split (2); total hip replacement (1); mastectomy/axillary lymphadenectomy (1); breast reconstruction following mastectomy/axillary lymphadenectomy (1); Extracorporeal Membrane Oxygenation (ECMO) (1); port at infraclavicular region (1); prolonged lumbar surgery (1); restraints for a combative patient (1); subclavian artery access in the infraclavicular region (1); thoracic outlet syndrome surgery (1).

**Table 3:** Localization of Brachial Plexus Injuries.

Metrics	Number of Cases (*n* = 52)
Trunk	22 (42.3%)
Upper	5 (22.7%)
Lower	17 (77.3%)
Cord	12 (23.1%)
Medial	4 (33.3%)
Lateral	4 (33.3%)
Posterior	4 (33.3%)
Pan-plexopathy	10 (19.2%)
Combinations	8 (15.4%)
Median cord and median nerve	2 (25.0%)
Upper and middle trunks	1 (12.5%)
Lateral and posterior cords	1 (12.5%)
Medial and posterior cords	1 (12.5%)
Lateral cord and median nerve	1 (12.5%)
Median cord and median nerve (R)	1 (12.5%)
Pan-plexopathy (B)	1 (12.5%)

B: bilateral; R: right.

**Table 4:** Mechanism of Brachial Plexus Injuries.

Metrics	Number of Cases (*n* = 52)
Stretch	48 (92.3%)
Direct *	4 (7.7%)
Compression **	1 (3.8%)

* The four direct injuries were the following: thoracic outlet syndrome surgery (1); port insertion in the infraclavicular region (1); subclavian artery access in the infraclavicular region (1); Extracorporeal Membrane Oxygenation (ECMO) (1). ** The one compression case was of the median nerve in a patient who also had an ipsilateral stretch injury of the medial cord of the brachial plexus.

**Table 5:** Electrodiagnostic Findings of Brachial Plexus Injuries.

Metrics	Number of Cases (*n* = 52)
Reduced motor unit recruitment and/or increased polyphasic units	48 (92.3%)
Absence of or low amplitude sensory nerve action potentials	48 (92.3%)

**Table 6:** Measures to Decrease the Likelihood of an Iatrogenic Brachial Plexus Injury.

Arm abduction < 90 degrees in supine patients with the hand and forearm kept in full supination. Avoid arm abduction associated with head rotation to the contralateral side. Avoid prolonged combined abduction and external rotation. Use of protective padding and padded arm boards and tucking the arms at the patient’s side in spinal surgeries. Limiting over-lowering the center of rotation during shoulder surgeries. Reducing the opening degree of the sternum, caudal placement of the sternal retractor, constant traction on the sternal halves, and avoidance of asymmetric traction are recommended during cardiac surgeries with a median sternotomy. Intraoperative neuromonitoring: SSEPs may indicate if the nerves are stretching beyond normal thresholds which may result in peripheral nerve ischemia. Regular assessment during prolonged surgical procedures to minimize the risk of iatrogenic injury. Postoperative neurological evaluation and EDX studies to promptly detect and treat potential nerve lesions.

## Data Availability

All of the data for this study is included in the current article.
